# “I Grew Up Amidst Alcohol and Drugs:” a Qualitative Study on the Lived Experiences of Parental Substance Use Among Adults Who Developed Substance Use Disorders Themselves

**DOI:** 10.3389/fpsyt.2022.768802

**Published:** 2022-02-02

**Authors:** Florien Meulewaeter, Elisabeth De Schauwer, Sarah S. W. De Pauw, Wouter Vanderplasschen

**Affiliations:** Department of Special Needs Education, Faculty of Psychology and Educational Sciences, Ghent University, Ghent, Belgium

**Keywords:** substance use, intergenerational, adult children, stigma, lonelineness, trauma, childhood, adulthood

## Abstract

Experiencing parental substance use (PSU) has been associated with a heightened risk of developing substance use disorders (SUDs) in offspring. The primary goal of this study was to explore perspectives of adult children with lived experience of PSU who also developed SUDs themselves through first-hand experience. This study was conducted in Flanders (Belgium). A qualitative exploratory research design was applied. Seventeen semi-structured interviews were conducted with adult children of parents with SUDs (range: 29–48 years) who themselves had developed SUDs. All interviews were audio-taped and transcribed verbatim. Three overarching themes emerged through thematic analysis: 1) loneliness and neglect in childhood; 2) stigma and the self; and 3) the role of social connection in substance use and recovery. The narratives highlighted the central role of feelings of loneliness, isolation and belonging among children of parents with SUDs in childhood and adulthood. Increasing public awareness on the impact of PSU on children and accessible support is needed to overcome stigma and remove barriers to social inclusion for children of parents with SUDs. Findings may prove valuable in informing policy, program and treatment development aimed at breaking maladaptive intergenerational cycles.

## Introduction

Generational continuity in the use of substances has gained attention for several decades (1–4). There is growing evidence showing that several biopsychosocial factors may contribute to heightened substance use risk in children of parents with substance use disorders (SUDs) (5–7), indicating that both genetic (8–10), and environmental factors (11, 12) play an important role in the heightened susceptibility to and manifestation of SUDs later in life (13–15).

Growing evidence shows an association between early life stress and adversity and heightened risk for developing SUDs in adolescence (16–24), which is a critical window for initiation, experimentation, and establishment of more regular patterns of substance use (25–32). A substantial proportion of the relationship between childhood adversity and substance use in adolescence is mediated through individual, interpersonal and community factors (33, 34). Evidence suggests that associations between cumulative stressors and adolescent substance use are moderated or mediated by genetic factors (e.g., related to cortisol regulation) (21, 35, 36), intrapersonal factors (e.g., executive cognitive function, self-esteem, impulsive behavior, inhibitory control, temperament) (37–41), or interpersonal factors (e.g., household and peer influences) (42–51).

Experiencing parental substance use (PSU) has been particularly associated with a heightened risk of developing SUDs in offspring (3, 52–57). Since PSU may impede parenting and the provision of a nurturing environment (58–60), the role of environmental and household stressors in the heightened risk of substance use continuation across generations has increasingly gained attention, with a particular focus on parental influences (e.g., parenting skills, parental inhibitory control, parental monitoring and discipline, parental modeling, harsh parenting) (42, 56, 61–65).

### The Current Study

The last decades, there is a growing interest for the group of “adult children” from various dysfunctional family backgrounds. To date, only a small body of qualitative research has explored perspectives of adult children that have been raised in a context of PSU. The primary goal of this study was to explore perspectives of adult children with lived experience of PSU who developed SUDs themselves through first-hand experience. When we refer to “adult child,” we are referring to a person who is currently 18 years of age or older, asking them to reflect on their past parent-child relationship. Our findings are intended to engender a deeper understanding of adult children's lived experiences of growing up with PSU that can inform both public perceptions and clinical and public health efforts to support families with PSU. This study addresses the following research questions:

- How do adults who have SUDs reflect on their upbringing in a context of PSU?
- How do adults who have SUDs reflect on pathways toward substance use initiation and early recovery?

## Methods

The present study is nested in a broader qualitative doctoral study at Ghent University (Belgium) on growing up in a context of PSU. Face-to-face semi-structured interviews with 46 adult children who were raised in a context of PSU were conducted, of which 17 had developed SUDs themselves. Only the interviews conducted with these 17 participants are analyzed here. Ethical approval was granted by the Ethics Committee of the Faculty of Psychology and Educational Sciences (Ghent University) (EC Reference 2018/42). Before interviewing, oral and written informed consent of the participants were obtained. Consent to digitally record interviews was granted and participants were informed that only the interviewer (first author) would have access to the recordings. Participants participated in the study on a voluntary basis. Names and potentially recognizable features have been altered or omitted to protect participants' privacy.

### Context of the Study

This study was conducted in Flanders, the Dutch speaking northern part of Belgium and a highly populated urban area (~6.5 million people). In recent years, Flanders has been faced with an increasing prevalence of drug use, with alcohol (licit), cannabis, cocaine, and ecstasy (illicit) being the most prevalent substances (66). In Belgium, the social cost of licit and illicit substances is estimated to be considerably high (67). Individuals with SUDs are characterized by high health care needs and use of health care services in Belgium (68), affecting individuals and their families adversely.

At the time the participants of this study were children (±1970–1990), the health care services available to individuals with SUDs fell under federal jurisdiction. The geographical spread of alcohol and drug services was limited, with a total absence of a family perspective in treatment systems. Resources for child and youth care services were also scarce and addiction in the family was either overlooked or strongly condemned, leading to the separation of children and their parents. Since the last decade, the Federal Government has conferred on powers to The Flemish Community, which is currently the competent authority for issues relating to social welfare and mental health care. Since this transfer of powers, the Flemish government puts increased emphasis on recovery-oriented and community-based services and care as an alternative to institutionalization (69, 70). In recent years, the government gradually upscaled funding for substance use prevention and treatment services, including outpatient and inpatient programs for parents with SUDs and children—albeit limited—(71–73), and harm reduction initiatives (74).

In spite of increased financial or other operational resources for prevention and treatment programs targeting individuals with SUDs in Flanders, parenting while experiencing SUDs remains to be a highly stigmatized condition (64). What's more, parental substance use often has a powerful impact on decisions about termination of parental rights (75). Child and youth care services tend to approach mental health conditions related to SUDs with the primary intention to protect children from harm, which is reflected in lack of interagency collaboration between substance use treatment agencies and child and youth care services. Recently, an increasing demand for developing legal measures that can protect unborn children from prenatal and preconceptual harm arose, especially in the context of maternal substance exposure. In the margins of a broader international tendency toward governmental involvement relating to pregnancy and substance use (76–79) and in the absence of a legal regulatory framework, legislative proposals and decrees on prenatal protection are currently debated. These proposals are primarily aimed to impose mandatory decisions on unborn children (e.g., out-of-home placement prior to giving birth) and forced admission to inpatient treatment (80).

### Study Design and Data Collection

Given the relative lack of prior published studies on the perspectives of adult children who were raised in a context of PSU in Flanders and internationally, a qualitative exploratory research design was applied (81). This allowed the researcher to explore a topic with limited coverage within the literature and allowed the participants of the study to contribute to the development of new knowledge in that area. The current research is one of the first studies to explore the experiences of adult children who were raised in a context of PSU, focusing on childhood as well as adulthood experiences on substance use.

Data were collected between November 2018 and December 2019. A purposive sample was selected to study this topic in-depth. Adult children were recruited through two outpatient and three inpatient substance use treatment services in urban and inner-city areas in Flanders. Eligibility criteria included being aged 18 years or older, having experienced PSU as a child, and having received a SUD diagnosis according to DSM-5 criteria (82). This diagnostic status was established through clinician's written reports and verbally confirmed by the participant to the interviewer. Potential participants were given written and verbal information about the study by the interviewer or a member of staff at the recruitment service and, if they expressed interest, were offered the opportunity to participate in an interview at a time convenient to them. Participants were informed they were contributing to a study on the experiences of growing up in a context of PSU. All interviews were conducted either in a private room at the service from which they were recruited or in a quiet local café, by the first author (FM). The average duration of the interviews was 109 min (range: 66–197 min). All interviews were audio-taped and transcribed verbatim in participants' and interviewer's same Dutch native language, by which all interviews were conducted. The interviewer transcribed the interviews during the data collection, which enabled her to check the quality of the data (83).

#### Participants

The participants were 17 adults (range = 29–48 years; mean = 35 years), all of whom had one or both biological parents (one stepparent) either actively addicted or in recovery from a drugs (*n* = 9) or alcohol (*n* = 8) addiction (Table 1). Twelve participants identified as male, five as female, and all identified as White. Participants' educational levels varied, all had completed primary school, the minority had completed college or university (*n* = 3). All participants had received a SUD diagnosis according to DSM-5 criteria, and reported receiving current (*n* = 14) or prior (*n* = 3) substance use treatment episodes, trying to maintain early recovery (stabilized substance use or cessation). None of the participants reported having experienced prenatal alcohol or drug exposure. Of the eight participants with parents with drug use disorders, seven were male. Eight participants reported current (on probation; *n* = 2) or prior (*n* = 6) incarceration. Three participants said they were in a current relationship and seven had children. Polysubstance use was common in both participants and their parents. Cocaine, amphetamine and heroin were among the most reported primary substances of choice, next to alcohol.

**Table 1:** Participants' characteristics.

**Characteristics**	**Number**
	**(*N* = 17)**
**Adult children**	
Gender	
Male	12
Female	5
Age	
28–34	7
35–40	8
41–48	2
Primary substance of choice	
Alcohol	1
Illicit drugs	16
Age substance use onset	
Early onset (5–9 years)	5
Early-mid onset (10–15 years)	8
Mid-late onset (16–18 years)	4
Current treatment	
Outpatient	4
Residential (on probation)	8 (3)
Prison-based program	2
Prior treatment	
Outpatient	1
Residential	2
Current incarceration	2
Prior incarceration	6
Subjected to child protection proceedings as a child	3
**Parents**	
Gender of parent(s) with SUDs	
Father	11
Mother	1
Both	5
Primary substance of choice	
Alcohol	9
Illicit drugs	8
Reported specialized substance use treatment admission in one of the parents	2
Participation in self-help groups	2
Prior or current incarceration	6

Information about parents was collected by family history information from the respondents. The majority of participants reported having lived in a socially unstable situation during childhood (e.g., family living arrangement, economic hardship, housing instability, parental mental health conditions). Four participants referred to frequent relocations during childhood. Six participants reported having experienced extreme poverty during childhood. Employment status among parents varied from both paternal and maternal unemployment (*n* = 2) to different kind of jobs that parents tended to hold. When parents were employed, respondents often reported isolation in parents outside of work. Only two participants reported episodes of parental substance use treatment. Five participants reported having experienced parental incarceration during childhood.

#### Interview Guide

Semi-structured open-ended questionnaires were used as method for data collection. At the start of the interview, each participant was asked a short series of demographic questions (such as age, family composition, what type of substances parents used,…) to enable the interviewer to construct a genogram to understand the family structure. The remainder of the interview was then semi-structured in format, and started with a broad question asking participants to describe general feelings and experiences related to their childhood. The interview guide used during each interview was honed to probe for participants' recollection of childhood experiences related to PSU and perspectives on pathways to substance use initiation and early recovery. Six primary open-ended interview questions guided each interview (Table 2). At the end of each interview, participants were invited to contribute any information they felt was important but had not been covered in the interview. A timeline approach was adopted throughout the interviews as an integral tool for structuring the complexity of participants' experiences (64, 84). This visual approach was also used to increase respondents' control in directing the interview, acting as a middle ground between the interviewer and the respondent (85).

**Table 2:** Interview guide (simplified version).

**Engagement question**	**Could you tell me a bit more about how you experienced your childhood?**
Exploratory questions	- Could you tell a little bit more about how you experienced family life as a child? - Have you ever received support related to your needs as a child? - How old were you when you first started using substances? - Can you describe in what context you started using substances? - Did any particular event in your life serve as a major turning point that caused you to enter treatment? - What factors have enabled or hindered your own recovery process in adulthood?
Exit question	Is there anything else you would like to share that is important to you as being a child of parents with substance use disorders?

### Data Analysis

Data analysis was initiated soon after data collection, allowing the authors to become more aware of emerging categories and themes (86). Using Braun and Clarke's (87) six phase thematic analysis framework for qualitative data analysis, the transcribed data were inductively analyzed. First, all transcripts were subjected to a “first reading” with comments written in the margin. Initial *in vivo* coding was then used to summarize data segments line-by-line (e.g., “feeling isolated”), followed by a second round of axial coding to disaggregate data into broader analytic terms and to define relationships between initial codes (e.g., “substance use to cope with isolation”). Initial and axial codes were used to conceptualize the data by summary and to help condense content for comparison between transcripts. After coding, potential subthemes were identified (e.g., “continued substance use as a fulfillment of relational needs”). From these subthemes, many were combined to form overarching main themes (e.g., “the role of social connection in substance use and early recovery”), whereas others that were less representative were discarded (e.g., “sensation-seeking”). These main themes were repeated throughout the data set and represent the most widely shared experiences that adult children perceived as being central to their childhood and adulthood experiences. After identifying three main themes, related quotations from each transcript were reviewed to ensure that the final themes accurately represent the patterns observed. Finally, the themes were defined and refined by selecting and further analyzing quotes that exemplify each theme within the context of each case. Refinement and clarification of themes occurred collaboratively among research team members until we solidified these themes and excerpts. Quotes have been translated to English by the first author. We stayed as close as possible to the literal translation and errors specific to the native language in which the interviews were conducted, have been corrected to aid readability.

## Results

The primary aim of this analysis was to explore adult children's childhood experiences with PSU and their own experiences of substance use initiation and recovery. The analytic process resulted in the identification of three major themes: “loneliness and neglect in childhood,” “stigma and the self,” and “the role of social connection in substance use and recovery.” Each of these themes contains multiple subthemes, which will be discussed in more detail below.

### Theme 1: Loneliness and Neglect in Childhood

#### Loneliness and Social Connection

When respondents reflected on their childhood, the most frequently reported theme was the feeling of being completely alone as a child.

*Because of the addiction of my parents, I never had a family. I also had a hard time having friends who accepted me, it made me feel alone a lot. Even today. (Nora, 28 years old)*

Some respondents expressed feelings of humiliation because of experiencing PSU. Saraï revealed:

*As a child, I used to feel alone a lot, because you always have to hide something. Your parents are drug addicts, you can't say that to anyone. You feel humiliated [pause]. In many ways. (Saraï, 36 years old)*

*All participants expressed having experienced shame about their rearing environment as a child*.

*I was always in-between like ‘this is just normal' and ‘I hope no one will ever know', because I did not wanted to be known as (–) [pause]. There is a shame. (Raul, 35 years old)*

*Reaching out for support as a child was reported to be shame-inducing and evoked feelings of guilt toward parents. As a result, children avoided disclosure about their childhood experiences and stressors present in the home. In some cases, this perceived stigma caused whole families to be isolated from the outside world or supportive others. Scott described how his mother was reluctant to help and how he feared professional involvement as a child:*

*She pushed them [professionals] away, she didn't see her drug usage as a problem. Professionals had no control over it. (…). As a kid, I didn't say much either [pause]. That could have led to misery (…). It's me who had to go home every day. (Scott, 29 years old)*

One respondent stated how he “did not dare to talk to care providers or teachers, because of serious threats from his father about what would happen” if the child disclosed. Even when problems were obviously impacting children severely according to respondents' narratives, they felt that relatives or professionals took no further action, leaving them feeling alone and sometimes betrayed as a child. Most of the respondents reported that their parents never reached out for help either, which resulted in perceived social isolation.

#### Childhood trauma and neglect

Narratives revealed that stressful environmental factors, including emotional and material neglect, endangered several respondents' physical and mental health as a child. Almost all respondents expressed a lack of parental warmth, with some respondents referring to “being neglected or abandoned.” Four respondents disclosed to be sexually abused as a child. Physical abuse was also frequently reported.

*Beating me and giving those strange punishments… Locking me up in the bathroom all day, without light, without food, ehm… [pause]. Or having to sleep on the floor, and she… I mean, me on a tiled floor (…), while she was lying on a mattress, ehm… (Scott, 29 years old)*

The vast majority of the respondents reported childhood chronic stress. This stress primarily stemmed from unpredictability and insecurity in the home environment, parental unavailability, parental violence, and financial or material hardship.

*A parent who uses drugs usually isn't there, doesn't like to take drugs in front of children, goes somewhere else. My parents had no source of income and had to fetch their drugs everywhere. (Saraï, 36 years old)*

Several respondents pointed out to have been used for obtaining and providing for parents' alcohol or drugs. Home environments that were characterized by parental drug use, generally held more links with poverty (e.g., no coverage of basic needs, being consistently food insecure), witnessing household substance use (access to drugs in the immediate social environment), exposure to drug-related activities in the home (drug dealing and consuming), parental incarceration or criminogenic activities, or experiencing parental drug withdrawal or overdose. The long-lasting impact of childhood trauma appeared from the narratives, which often resulted in mental pain that lingered into adulthood.

*I feel like I keep returning to it [adverse childhood experiences] [pause]. And when I sleep, I dream about it, everything comes right back… so I wake up with it everyday. (Christoph, 43 years old)*

The majority of respondents indicated ever having experienced suicidal ideation and four respondents spoke about attempted suicide. Several respondents expressed resentment toward their parents but also toward the wider society for not having received the needed support during childhood. Saraï, who found her mother after a heroine overdose when she was 8 years old, reported:

*I've realized that everything I'm in now, has revolved around my family. That pain, losing everything,… [pause]. My destructive behavior originated from it. (…) I've always used to feel so alone. (…) You get mad at society: ‘I've been maltreated as a child, and you didn't do a thing'. (Saraï, 36 years old)*

### Theme 2: Stigma and the Self

A second important theme that emerged through the thematic analysis revealed how respondents' upbringing had contributed to a lack of positive self-esteem and interpersonal difficulties. This influenced how respondents navigated the social environment beyond the home, which further influenced perceptions of social isolation and stigma.

#### Self-esteem and interpersonal connection

Narratives revealed how self-doubt and low self-esteem resulted from respondents' childhood experiences. Because of confusion about what happened in the home environment, several respondents reported to attribute household problems or parental behaviors to themselves as a child.

*By all those factors… I thought as a child: ‘Indeed, I'm failing… I'm a monster… I'm bad'. Those were my beliefs. And they still are. Because… why do such bad things happen to me? (…) I felt like a burden. (Astrid, 33 years)*

Due to familial secrecy and shame around PSU, several respondents experienced feelings of alienation.

*At some point, I went to school, and I started to feel myself different from other people. The other children could count on their parents if anything happened; we didn't have that. (Saraï, 36 years old)*

The narratives elicited how all respondents had, each in their own different way, developed internal or external problem behaviors that stemmed from their upbringing.

*I'm functioning and no one would ever think that I had a problem, but… [pause]. Inside, having this minus one level of self-worth… I think this opened a nice door for drugs, because then you can escape and then you can feel happy, chemically happy. (Jenny, 39 years old)*

Difficulties with interpersonal connection was a frequently reported theme throughout the interviews. Some respondents mentioned how, due to being devaluated as a child, they psychologically distanced from others to avoid further negative or painful experiences. This in turn impacted close relationships from childhood through adulthood, and sometimes led to social isolation.

*Nobody knows what was going on in childhood. You are always playing scene, you're never your true self. In order not to show to the outside world that you're different, you try to assimilate. Always hiding, hiding yourself, hiding your feelings, hiding everything. (Christoph, 43 years old)*

#### Navigating the Social Environment Beyond the Home

Several respondents expressed a lack of understanding about their childhood in their social environments. They pointed out a lack of awareness in society about the impact of childhood experiences through adulthood and a lack of openness to talk about it.

*If I had to say that to other people in society, how will they think about it? That's exactly what I want to emphasize. That's exactly what you're ashamed of [as a child]. You cannot (–) If I were to say that to someone who's employed, or who is practicing everyday… I cannot talk about it, they do not understand the situation I'm in now [pause]. Preconceptions originate from it, but sometimes you just wish you could say that. (Scott, 29 years old)*

Respondents pointed out that schools could have a greater role in intervention and support for children of parents with SUDs. Several respondents mentioned that schools should instill a culture of awareness about the impact of PSU on children, and establish a climate in which children feel safe to have a conversation with a trusted adult. Respondents emphasized the value for children of parents with SUDs of being offered opportunities to talk, and having a trusted adult or a support network that extends through childhood, where children can talk openly about sensitive subjects such as PSU.

*As a child of addicted parents, above all, I think it's important to keep communicating. You shouldn't think: ‘I'm feeling down, I'm going to be a burden'. (…) Not cutting yourself off, I think that's important (…). In the past, I always used to be on my own (…). If children know that they can talk about it and that they‘re not in it alone, I think it can really help. (Nora, 28 years old)*

*I wish I've had a mentor. (…) Someone I could lean on. A father figure, a teacher who's designated (…). I missed that. Getting attention, getting affection. Someone who won't let go of me and does say so: ‘I won't let go of you'. (Scott, 29 years old)*

Respondents reported they grew up in an environment where they have often seen their parents using alcohol or drugs as an answer to problems (e.g., relational, financial, judicial), which one respondent referred to as “an environment not being fostering to further development and choices made later in life.”

*As a child, you automatically end up in that kind of environment too. When you grow older, you start to notice: you have made certain decisions, stupid decisions, you are in debt, you are in poverty… you automatically meet these same people too. (Titus, 31 years old)*

Several respondents stated that they always ended up with people that also used drugs, in one way or another, or that they appear to find and seek others with similar inclinations.

*We had to relocate a lot during childhood. I always had to make new friends. That wasn't always easy, but each time when I met someone, it was guaranteed a user. It must have been written on my forehead. (Raul, 34 years old)*

Through data-analysis, it became clear that childhood trauma, financial hardship, social isolation, and substance use often ran across generations. The feeling of “being stuck in an endless cycle of disadvantage” regularly popped up from the narratives. Respondents framed the difficulties they had encountered or still are encountering in life (e.g., financial or employment difficulties), as a recurring circle of “*not contributing to society”* that they found (or still find) hard to break. Scott referred to the impact of addiction on multiple life domains.

*In such a situation, when the parents have never worked, it is more likely that the child will not work either… Because they never seen it at home, because they don't know how to apply [for a job]. [pause] It's not just the addiction itself, it's everything around it. [pause] And that's what costs society money. (Mauro, 45 years old)*

### Theme 3: The Role of Social Connection in Substance Use and Recovery

All respondents had developed SUDs throughout their life and were in the early stage of recovery from SUDs at the time of the interview. Without assuming that motivation for substance use initiation and recovery arises solely out of environmental or interpersonal influences, the specific role of social relationships in substance use initiation, continued substance use, and recovery stood out from the narratives and will be further detailed below.

#### Substance Use initiation

##### Parental Influence

The majority of the respondents reported they first came into touch with alcohol or drugs in the home environment. Hence, substance use initiation among respondents was frequently found to be explicitly or implicitly entwined in the nature of parent-child relationships. A first striking dynamic according to the narratives, was parents' active providing of alcohol or drugs.

*I grew up amidst alcohol and drugs. My father was a drug dealer. When I was thirteen, my father was busy at the table one night. I saw white powder everywhere. I wondered ‘what is that?', he said ‘get your finger wet, I'll show you how to do it'. There was a tube lying there and he sniffed it. First, it was only in the weekend. But it quickly progressed toward a daily basis. When I was 15, I started basing cocaine, cleansing it with ammonia and put in on a pipe. (Neil, 33 years old)*

Because respondents had frequently been exposed to alcohol- or drug-related activities in the home environment from an early age and considered it to be “normal” and part of everyday life, their tolerance toward the perceived level of seriousness of risks (e.g., consumption methods) and the degree of substance use severity they set for themselves was reported to be higher. They normalized or minimalized their own substance using behavior.

*When I did that for the first time, I was aware that it was wrong, but still I did it. However, I've also seen what drugs [heroin] my father and stepbrothers were taking, so I thought it couldn't get any worse than that: ‘it's just amphetamines and cocaine', but that's just as bad of course. (James, 38 years old)*

Some respondents had the feeling that drugs were deployed by parents as a means to forging a relationship with their child, since parents came to realize that drug-related activities were facilitative for regulating a parent-child relationship.

*I think my dad gave me drugs, just to establish a friendship. He didn't do that from a father position, I think, but to having at least something together. I don't know why I didn't understand it was wrong. (Raul, 35 years old)*

Several respondents reported that they had developed a drug-mediated relationship with their parents throughout adolescence.

*I was aware that when my father was in the kitchen with a closed door, I wasn't allowed to enter. I've never seen my father sniff a line. But when I was fourteen, I also started using, together with my father. (Naomi, 36 years old)*

*There has never been father-son-bonding between us. Only drug-bonding. (Neil, 33 years old)*

Another striking dynamic according to the narratives, was children's early curiosity. Due to parents' habituated behavior and alcohol or drugs preoccupation, some children reported they became curious about its effects, which lowered the threshold to start using.

*I often threw my mom's stuff away, and I've often asked her to quit. Eventually, I started wondering why she couldn't quit. (…) I'm not going to blame her that I started using, but I did wonder why she couldn't stop, and what was at the root of it. (Naomi, 36 years old)*

Some respondents reported that, as a kid, they subconsciously copied their parent's drug using behavior.

*When I was 6 years old, we received complaints from teachers. At noon during lunch, crumbs of my bread lay on the table. I gathered those crumbs together, and started sniffing them with a straw. (Philip, 39 years old)*

##### Peer Influence

While some respondents reported parents to be a major contributor to substance use initiation, others associated substance use initiation with peer involvement. Respondents often had to grow up quickly, which was for example due to having to rely on oneself from a very young age, or permissive parenting. As a result, respondents were given a lot of freedom from an early age, and ended up on the street quite early, where they encountered (mostly older) peers. Many reported to have quickly been engaged in “wrong environments.”

*I was home alone every weekend (…). I started smoking weed with friends from an early age. I started to hang out on the street when I was 14-15 years old. I immediately hang out with guys aged 22-23, never with same-aged peers. That's also why I started cocaine so early, those guys were already on it… When I was 16, I was already committing robbery, and I was already engaged in extortions. (Titus, 31 years old)*

Respondents mentioned different peer settings where they encountered substances (leisure, school,…). Often, it started with being offered only one pill, or only using during the weekend; but this quickly progressed into more intensive use.

#### Continued Substance Use as a Fulfillment of Relational Needs

Interviews revealed how continued substance use fulfilled several relational needs, of which the most outstanding were the need for belonging, and coping with loneliness and isolation. As such, continued substance use was marked by coping with underlying affective difficulties, in which “the need for belonging” served as an enabler of continued substance use in the face of isolation. Respondents reported about continued substance use in terms of a change from an enjoyable activity with peers, feeling comfortable and self-confident being in the company of other people when using, and how it the longer the more led to isolated and more risky substance use behavior.

The most reported function of substance use among respondents was to have a sense of belonging and to expand social bonds, since the use of substances increased the ability to talk, personal openness and self-confidence.

*It allowed me to get to know a lot of people. (…) In the longer term, I had a lot of friends, and I could turn to everyone. (Philip, 39 years old)*

Several respondents reported having a hard time finding an identity in the world in adolescence and emerging adulthood, since childhood was often only about “survival,” with their later social competence being put in jeopardy. Sometimes, the difficulty in establishing and maintaining connections with others led to susceptibility to and increased need for using substances.

*I had finally found something to be more social, which was mainly the reason for my drug use... I usually don't say a word, I'm often shy, and with drugs, alcohol, ecstasy, cocaine… you can just chat with everyone. (Titus, 31 years old)*

Less relationship reward had been available to respondents during childhood and adolescence, which often drove them back to substances, which from respondents' narratives appeared to function as a relational substitute. Using substances helped several participants to feel the pleasure and the basic need of connectedness and personal fulfillment. However, this “habit” further isolated them.

*Talking about my feelings as a child? I took my usage [pause]. My best friend [pause]. It didn't say anything back... It didn't comment... It always made me happy… It always made me feel good [pause]. Drugs were my best friend. (Neil, 33 years old)*

Interviews elucidated that substance use also served as a response to early stressors associated with the home environment. On the longer term, continued substance use had in several participants the additional function of coping with negative early life experiences and stress derived from childhood trauma.

*My usage was more like a way out… Stress was building up all the time and I had to let go of that stress, but I couldn't be released of it... The stress we used to live in... Because parents who are drug addicts, they lose everything after a while. They lose their house, bailiffs come in… The worst things happen to you as a child. (Saraï, 36 years old)*

As the substance use intensified, the function of substance use evolved from rather a “social activity” and being able to form relationships, to “using alone” (e.g., because friends were not there during the week), which led to increased isolation. Respondents mentioned that in response to that isolation, alcohol or drug use helped them cope. This cycle led to physical or psychological dependence over time, whereby respondents indicated to “just need” the substances. This led to increased loneliness and isolation, whereby loneliness and substance use perpetuated each other.

*I didn't come out anymore, I had bad friends… I became completely socially isolated. Even though social contact has never been easy to me, you always want to belong somewhere… But after a while, you're not doing anything anymore, you can't keep your job because you get so tired… (Nora, 28 years old)*

*In the beginning it's fun, but it doesn't last. If you take it [cocaine] for a while, you don't come out anymore, you stay in your house, you hide yourself. I completely detached from people, I wanted to be alone. (Titus, 41 years old)*

Respondents stated that on the longer term, substance use led them to cope with psychosocial problems such as problems related to work and employment, family problems, social exclusion, depression, and suicide attempts. However, the fact that the usage fulfilled a particular need for belonging, made them persist. Consequently, respondents reported reduced motivation to engage in the pursuit of relationships. Instead, they increased substance use, which they themselves thought was becoming dangerous at some point.

*I've always been able to hide that I was addicted. I knew it about myself, but no one knew it around me. I was able to hide it all so well, because I did everything alone… buying from only one person and done. Nobody knew about it [pause]. You pretend to live a normal life, but in the end, you're actually feeding something very lonesome, something that's getting dangerous. And I realized it, but I didn't dare to do something about it. (Saraï, 36 years old)*

#### Behavior Change and Early Recovery

From the narratives emerged how social relationships functioned both as enablers and challenges to behavior change and early recovery. These processes of change appeared not to be linear “one-time success attempts,” but they rather were characterized by circularity between continuous contemplation, specific challenges and opportunities, and several endeavors. Respondents' motivation to change often originated from critical moments (e.g., hospitalization, overdose), which confronted them with the repetitiveness of patterns (“not wanting to walk the same path as my parents”). This opened up contemplation of treatment admission among several respondents.

##### Relational Enablers

Through analysis, it became clear how relational enablers could boost respondents' ability to successfully navigate challenges during stable periods of use and early recovery. Some respondents indicated that, what motivated them to treatment admission, retention, and early recovery, were positive role models being in sustained recovery, who kept the hope alive that “recovery is possible.” These persons were able to give advice to respondents based on their own lived experience of addiction and recovery. Some respondents drew strength from these persons, others indicated that they were looking for someone who had overcome addiction in the face of adversity.

*I've followed the example of my mother. She makes me believe that it's still possible… at any age. That it's never too late. My mom has a good life now, that's what I'm also striving for. (Kian, 35 years old)*

Among the respondents who had children themselves, most indicated that their own children were a motivation for recovery. Because respondents had never seen a good example, they wanted to approach things differently for their children. They wanted to be available as a parent, which leveraged their motivation for treatment. Also parent-child contact during treatment appeared to be a factor promoting treatment adherence.

*I want to be there for my daughter. Because I know, I've never had that myself. If she comes to visit now and she leaves, I have to put her in the car seat. She always says: ‘daddy, are you coming home with us?'. That's heavy. Then I say: ‘daddy first has to heal before daddy's coming back home'. (Raul, 34 years old)*

Throughout the narratives, the importance of the quality of the support from relatives emerged. Regular visits or contact and experiencing unconditional support was perceived to be highly meaningful and often impacted contemplation. The quality and unconditionality of relationships, especially through bad times (e.g., after being convicted, after an overdose), increased motivation for change.

*My stepfather came to visit me every day in prison. Every single day. Once he told me – I will never forget those words: ‘You've made a mistake, but even if you commit murder, you will forever be my son'. That's when my eyes really started to open: ‘now is the time to pick myself up again and tackle my addiction'. (James, 38 years old)*

Just as several respondents expressed hope from relationships with close relatives, two participants particularly drew hope from spirituality both as a basis for dealing with things from the past in a different way other than drug use, and in overcoming addiction vulnerability.

*Buddhism is what keeps me in the state of trying to be as controlled and as safe as possible. It helps me to redesign things in my mind. I need a very structured life and by practicing Buddhism, I can keep going. It helps me to more easily and more quickly answer to my vulnerability… like, waves will come, but… (Jenny, 39 years old)*

##### Relational Challenges

One of the most recurring challenges to recovery was parents and other close relatives' involvement in substance-related activities or active addiction (e.g., using or selling drugs). This often caused respondents to relapse after stable periods of use or post-treatment. Being alcohol- or drug-free for the first time (outside of a controlled environment), and knowing how and where to quickly get drugs, appeared to be a challenge for early recovery. It turned out that after periods of quitting drugs for a while, or after being discharged from treatment, parents were still facilitative for respondents' relapse into drug use.

*I just had quitted taking GHB for one week – right, one week isn't that long, but it is one week nonetheless. We agreed that he was just coming to visit me, but he brought me drugs. Of course I take it. (Naomi, 36 years old)*

After treatment discharge, respondents often had made the decision to break up with former negative interpersonal relationships and all the regular contacts they had before/during active addiction, aiming for sustained recovery on several life domains. Consequently, parents were often the first and only people they could turn to. Hence, respondents often ended up back at the “source,” where a lot of risk was involved. One respondent explained how he became involved in drug-related activities again after release from prison.

*I had quit using drugs at that time. But the moment I got out, I had no shelter and no one to turn to. I also had debts. My father said, ‘Titus, you can live with me on the condition that I can earn a lot of money'. (…) I've taught him how to sell cocaine, I had to teach him how to compose drug packages, how to deal with drug dealers, how much he was allowed to sell, … how everything works. (Titus, 31 years old)*

Another important impeding factor to early recovery that showed up from the interviews, were conflicted family relationships, since respondents' SUDs often had damaged family ties. As a result, respondents were unable to turn to family after stable periods of use or after treatment, because of shame (e.g., family members had found respondents after an overdose) and feelings of guilt toward their family (e.g., respondents threatened or put pressure on their families for financial purposes), which also arose from situations and accidents experienced during active addiction (e.g., hitting parents).

While the lack of a supportive social network led to relapse among several respondents, some respondents reported desperate attempts, due to the lack of a network where they could or dared to turn to, not wanting to be a burden to family members who already had been exposed to enduring stress.

*I tried to commit suicide twice, so that I was no longer a burden to my family. (…) I've used [drugs] for 23 years, those people have also suffered for 23 years. (Neil, 33 years old)*

Moreover, respondents did not want to go back to their old (substance using) networks after being discharged from treatment or after stable periods. Hence, some of the respondents ended up in isolation or homeless, which pushed them back into prior networks and substance using behaviors (e.g., using heroin as a remedy for fighting the cold), and served as an impediment to achieving sustained recovery.

*I was not allowed to go to my stepdad, because I had a very bad relationship with my family because of my [drug] use, so I ended up on the street. (…) I called my cousin, ‘can I come to you?', so I lived with him for a few months, but he was also a user. (…) I also lived in my car for a few months. (…) I had nothing left other than my car, so… (…) I sat around the table with my cousin, as we ran out of money. We started to commit thefts and burglaries throughout the country. We broke into all the schools to have money for drugs. (James, 38 years old)*

Also the relationship with the non-using parent or other relatives was often broken, so that respondents ended up in isolation, which lowered the threshold to substance use and suicidality.

*I was no longer allowed in my mother's house, the bond with her was completely broken. (…) All those friends around me, they were all [drug] users. Lost my job… (…) I thought I'd jump in front of the train. I was on the verge. I ultimately ended up in the emergency department with an overdose of drugs and medication. (Raul, 35 years old)*

A last important theme that emerged from the interviews was the sucking power of the environment that respondents were in during active addiction. This was an environment from which respondents could not easily withdraw, which often put pressure on early recovery processes. Detaching from negative peer influences after being discharged from treatment was often a necessary decision, but not an easy one which took a long time, since respondents were often still indebted to people from their (ex-)networks.

*They told me: ‘Titus, you know way too much'. I said ‘I'm never going to talk', I never did. (…) They have seen: ‘Titus does never talk, he did never betray us', and they said ‘Titus, It's all right, go live your life, go to your child, but don't come near us anymore; and don't do business, don't use drugs'. (Titus, 31 years old)*.

## Discussion

This study allowed a deeper understanding of previous work, by considering adult children of parents with SUDs' lived experiences of growing up with PSU in childhood and adulthood. Three themes were identified from the analysis: 1) loneliness and childhood trauma and neglect; 2) stigma and the self; and 3) the role of social connection in substance use and recovery. The implications of the findings will be discussed in the next section of the paper.

Our research adds to growing scholarly literature on the long-term impact of adverse childhood experiences (ACEs) and PSU on mental health and SUDs later in life (88–90). PSU has been associated with increased risk of offspring experiencing adversities during both childhood and adulthood (91, 92). Our findings contribute to the understanding of how PSU can cause a variety of harm to children, which may be related to unsafe family environment and long-standing stress (60, 93). Furthermore, this study showed how adult children faced loneliness and mental health stressors in childhood, and how this can contribute to mental health and substance use issues in later life (88, 91). Previous studies also pointed to the high burden and prevalence of multiple childhood adversities in children of parents with SUDs and in other substance using populations (94, 95). Therefore, this study highlights that preventive interventions for children of parents with SUDs constitute an important public health issue. Findings can contribute to shape the perspective of stakeholders, including the general public on growing up in a family with PSU. From this study, it is clear that early interventions that address common risk factors and multiple consequences of growing up with PSU should be available to children living with PSU and their parents. The role of relatives, teachers and professionals in the recognition and identification of children of parents with SUDs and to attend to their unmet needs by taking action (96) was emphasized. This study stipulated the need for increased social support that may buffer against negative consequences of exposure to PSU in childhood and adulthood.

This study provides insight in mechanisms that may contribute to feelings of loneliness, shame, social isolation, low self-esteem and lack of social support experienced by children of parents with SUDs (97–100). In this sense, the effects of secondary stigma became apparent for those relatives who are associated with stigmatized individuals, including parents with SUDs (101–105). It may be prudent to note that in this sample, parents did not receive substance use treatment during offspring's childhood and adolescence, what may possibly be explained by, amongst others, the impact of stigma and discrimination on parents' willingness to seek help and preventing access to treatment (103, 106, 107). However, stigma in families with PSU is constructed in relation to social norms (108, 109).

Further, this research pointed to the social stigma surrounding PSU. Given the disruptive effect of stigma on family cohesion in families with parental substance use, with relatives often experiencing to be “invisible” (99), this study emphasizes the need for applying a developmental and interactional perspective on social support to children and parents with SUDs, as stated by Newcomb ((110), p. 54): “Social support can no longer be considered strictly an external force impinging upon the individual; rather, it must be viewed as an evolving developmental and interactive process between an individual and his or her social environment.” Although children of parents with SUDs experience need for connectedness and receiving social support from trusted adults (97, 111), which has been shown to be protective in this group of children at-risk (112), feelings of shame and self-blame related to the upbringing may hamper help-seeking in children, with implications for long-term mental and physical health (104, 113, 114). Previous studies showed that also among parents with SUDs, other mental health difficulties often co-occur (115). Our study supports the need for systemic and stigma-reducing interventions to support children of parents with SUDs (60, 116, 117).

In line with Hoffman and Su (118), this study shows how parental substance use puts adolescents at significant risk of becoming involved in substance use and association with substance using peers. Peer substance use has shown to be more predictive for adolescent substance use, in comparison with parental drug use (47). Peer substance use and exposure to substance using friends have been strongly associated with adolescent substance use, both in offspring of parents with SUDs (119) and without SUDS (118). However, evidence is clear that the negative impact of growing up with a parent with SUDs on offspring substance use initiation and further progression toward SUDs is heightened when the child associates with substance using peers (119), what could be explained by wanting to receive validation and satisfy their need for belonging (120). Parental monitoring and family relationship quality indirectly predict later substance use by way of deviant peers (121). Deviant peer affiliation is clearly an important avenue for intervention when seeking to interrupt the intergenerational transmission of SUDs. Hence, findings underscore the importance of early assessment and intervention for peer relationships in adolescent offspring of parents with SUDs. Further, research points to the importance of conventionality (122) and quality (123) of peer relationships in mitigating adolescent SUDs risk. Given the fact that having fewer substance using peers has shown to be beneficial to adolescent substance use (122), and that the quality of adolescent peer networks predicts positive SUDs outcomes (124), promotion of prosocial bonding and peer groups, enhancing quality of peer relationships, and involvement in prosocial activities may be particularly salient for children with heightened genetic risk for developing SUDs (119, 123).

Recent studies found an association between loneliness in adolescence and health risk behaviors (125), including substance use (126). Given that this study points to the central role of feelings of loneliness, isolation and belonging in the progression toward SUDs, helping experience offspring of parents with SUDs human connectedness is an important part of prevention and intervention efforts. As the results show, offspring's attachment to substances fulfills underlying relational needs, serving as a substitute for human connectedness (127). As such, this study supports the hypothesis that SUDs represent, at least in part, a misplaced striving for connection [(127), p. 2]. Building prosocial support and qualitative interpersonal relationships may be an important mechanism for alleviating loneliness and may lead to decreasing substance use in an attempt to modulate loneliness and the need for belonging.

Finally, this study expanded research on SUDs and recovery by examining how interpersonal influences and social isolation affected substance use and recovery in adult children of parents with SUDs (128). In particular, this study improved understanding of how parent-child and other interpersonal relationships can both operate as a catalysator in the trajectory toward substance use initiation, as well as challenging in striving toward early recovery. Although families have been identified as a primary context of care for young adults' substance use treatment processes (129), this study showed that the family context among adult children of parents with SUDs is often not facilitative for their treatment and recovery processes. In line with evidence on the importance of relationships and social resources needed for initiation and maintenance of addiction recovery (130–137), this study confirms the importance of interpersonal relationships and social resources in readiness to change and early stages of recovery (131, 138–143). Although the importance of social networks in improving early and more sustained recovery outcomes has been established (144), this study provides insight in how family, parent and peer support systems can both inhibit and encourage recovery in offspring of parents with SUDs. Consequently, specific components of early recovery social networks, such as network size (133, 139, 145), quality (146), and density (138) have proven to be important factors in recovery outcomes. Strong associations have been found between ongoing contact with substance users and continued substance use (147). Contrarily, stronger identification with non-using groups, social networks including more people in recovery and fewer people in active use have been associated with improved treatment and recovery outcomes in emerging adulthood (133, 148–151). This study suggests the need to ascertain the exact nature of social networks and the social contexts within which they are developed (152). Prior to tailored treatment planning, the nature of social network support should be systematically assessed. Research provides additional evidence that many persons in substance use treatment possess non-substance using family or friends who are willing to support recovery efforts (153). Activating non-substance using family and friends has shown to provide potential pathways to help persons with SUDs access and benefit from community support (154, 155). Moreover, given that early recovery stages are sensitive periods for experiencing loneliness and social isolation (138), which has been associated with SUDs (156–158), adult offspring with SUDs may benefit from strategies to build and sustain prosocial connections and recovery-supportive networks (159), that prepare them for “normal everyday living” (137) and “outside living” in a way that promotes positive relational enactment.

### Limitations and Further Research

This study has contributed to a neglected area of research with regard to the lived experiences of PSU among adult children who developed SUDs themselves. However, limitations of this study have implications for the generalizability and validity of the findings. A first constraint is due to the study design. By relying solely on one-off clinical interviews, the study findings remain bound to the context in which this research is conducted. Future research is needed to validate the study findings by adopting structured survey assessment (e.g., standardized questionnaires). In addition, to understand the significance of the study findings, it is important to consider that this study was delimited by investigating only adult children of parents with SUDs' perspectives. Future research is needed to include parents' views to more closely examine the influence of specific factors, such as severity of substance use on caregiving and parent-child relationships. Also, due to a lack of information on parents' socioeconomic background, it is not clear how study findings may have operated differently across socioeconomic contexts. In addition, the data does not represent first-person accounts of direct childhood experiences, but of adults retrospectively reflecting on childhood experiences after having been in substance use treatment. This may have impacted both what participants viewed as relevant information to share during the clinical interviews, and also what can be understood as their own experience vs. what they have come to understand as a general experience of people who have parents who use substances. Another important limitation concerns the representativeness of the sample. This was a clinical sample of 17 mostly male, all-White adults from the Flemish Region. Future research is needed to know whether the views expressed are representative of this broader population. Finally, participants eligible for inclusion in this study were selected based upon the criterium of having a history of PSU. In the interest of understanding the specificity of vulnerability and protective processes, study findings need to be complemented by examining environmental protective factors and resilience among children with heightened genetic vulnerability who did not develop SUDs themselves (160–162). Knowledge of resilience factors may inform more targeted prevention and intervention efforts to optimize support for families experiencing PSU.

## Conclusion

This study investigated the lived experiences of PSU among adult children who also developed SUDs. A constellation of socio-relational and other environmental factors play a role in the intergenerational transmission of SUDs. Although these factors cannot be considered in isolation and need to be examined from an holistic biopsychosocial viewpoint, this study has illustrated that family, parent and peer environmental factors play a role in accounting for offspring outcomes; in particular, that environmental factors can influence the impact of high genetic risk regarding SUDs development in offspring. Children of parents with SUDs are at heightened risk for early stress, social isolation and developing SUDs, which, in the absence of adult buffering support, may affect adolescent and adult mental health. Social support and qualitative, prosocial relationships may contribute to prevent intergenerational continuation of SUDs over the lifespan. However, social support changes as a result of transactions between a person and his/her social environment, and must therefore be individually adjusted within existing constraints and contexts. Developmentally stable, positive and strong social bonds over the lifespan are of utmost importance for discontinuing the cycle of intergenerational SUDs. Therefore, reducing public stigma of SUDs in families and reinforcing and enhancing affected children's skills in persevering with help-seeking is imperative to foster a safe and nurturing family environment.

## Data Availability Statement

The raw data supporting the conclusions of this article will be made available by the authors, without undue reservation.

## Ethics Statement

The studies involving human participants were reviewed and approved by Ethics Committee of the Faculty of Psychology and Educational Sciences, Ghent University (EC Reference 2018/42). The patients/participants provided their written informed consent to participate in this study.

## Author Contributions

FM wrote the manuscript and was responsible for general ideas, conducted the interviews, made a first analysis, and drafted the manuscript. SD and WV designed the study. ED and WV provided feedback on the data-analysis process and substantially contributed to the final version and revisions of the manuscript. SD did editorial work on the entire manuscript. All authors approved the manuscript for publication.

## Conflict of Interest

The authors declare that the research was conducted in the absence of any commercial or financial relationships that could be construed as a potential conflict of interest.

## Publisher's Note

All claims expressed in this article are solely those of the authors and do not necessarily represent those of their affiliated organizations, or those of the publisher, the editors and the reviewers. Any product that may be evaluated in this article, or claim that may be made by its manufacturer, is not guaranteed or endorsed by the publisher.
